# Hidradenitis suppurativa and psoriasis: shared immunological links with metabolic-associated steatotic liver disease

**DOI:** 10.3389/fimmu.2026.1831174

**Published:** 2026-06-12

**Authors:** Wei Qiang Chng, Jake J. Liew, Choon Fong Liew, Mark Muthiah, Ming-Hua Zheng, Hazel H. Oon

**Affiliations:** 1 Department of Dermatology, National Skin Centre, Singapore, Singapore; 2 Hwa Chong Institution, Singapore, Singapore; 3 Specialist Care Group, Diabetes and Endocrinology Centre, Mount Elizabeth Medical Centre, Singapore, Singapore; 4 Department of Medicine, Yong Loo Lin School of Medicine, National University of Singapore, Singapore, Singapore; 5 National University Centre for Digestive Health, National University Hospital, Singapore, Singapore; 6 National University Centre for Organ Transplantation, National University Hospital, Singapore, Singapore; 7 MAFLD Research Center. Department of Hepatology, the First Affiliated Hospital of Wenzhou Medical University, Wenzhou, China; 8 Key Laboratory of Diagnosis and Treatment for the Development of Chronic Liver Disease in Zhejiang Province, the First Affiliated Hospital of Wenzhou Medical University, Wenzhou, China; 9 National Skin Centre and Skin Research Institute of Singapore, Singapore, Singapore; 10 Lee Kong Chian School of Medicine, Nanyang Technological University, Singapore, Singapore; 11 Yong Loo Lin School of Medicine, National University of Singapore, Singapore, Singapore

**Keywords:** biologic, fatty liver, glp-1, hidradenitis suppurativa, inflammation, metabolic dysfunction-associated steatotic liver disease, obesity, psoriasis vulgaris

## Abstract

Chronic inflammatory skin conditions such as hidradenitis suppurativa and psoriasis vulgaris exhibit a significantly elevated prevalence of metabolic dysfunction-associated steatotic liver disease (MASLD), with studies indicating rates as high as 57% among affected individuals. This narrative review explores the underlying immunological mechanisms that connect HS and psoriasis with MASLD, emphasising the role of chronic systemic inflammation, immune dysregulation, and shared immunologic links between the diseases. This article also urges for enhanced awareness among dermatologists regarding the potential pharmacological interventions for patients with concurrent HS or psoriasis and MASLD, including glucagon-like peptide-1 receptor agonists and biologics, while acknowledging the need for further research to elucidate the efficacy and safety of these treatments.

## 1 Introduction

Multiple studies have demonstrated that patients with hidradenitis suppurativa (HS) and psoriasis have a higher prevalence of MASLD compared to matched controls, with prevalence as high as 57% even after adjusting for metabolic risk factors like obesity, hypertension, and hyperlipidaemia (1–3). Chronic systemic inflammation in HS and psoriasis contribute independently to hepatic steatosis through various immunological mechanisms centred on shared mechanism of innate and adaptive immune dysregulation, common epigenetic mechanisms and adipokine imbalance. In this narrative review, the authors synthesise the immunological mechanisms behind the link between HS and psoriasis with MASLD, thereby establishing a skin-liver axis.

MASLD, formerly known as non-alcoholic fatty liver disease, is a major health burden, affecting approximately 30% of the entire population (4). As “non-alcoholic” inaccurately reflects disease aetiology, an international panel updated the nomenclature to reflect the role of metabolic dysfunction in contributing to hepatic steatosis. These include MASLD, alcohol-associated liver disease (ALD), metabolic dysfunction and alcohol-associated liver disease (metALD) and other rarer causes such as Wilson’s disease (5). **Table 1** summarises the criteria for diagnosing MASLD.

**Table 1:** Criteria for MASLD.

Cardiometabolic adult criteria (at least 1 of 5) in the absence of secondary causes of steatosis 1. Body mass index ≥25 kg/m^2^ [23 for Asians] or waist circumference >94 cm (male)/80 cm (female) OR ethnicity adjusted 2. Fasting serum glucose ≥5.6 mmol/L [100 mg/dL] OR 2-hour post-load glucose levels ≥7.8 mmol/L [≥140 mg/dL] OR HbA1c ≥5.7% [39 mmol/L) OR type 2 diabetes OR treatment for type 2 diabetes mellitus 3. Blood pressure ≥130/85 mmHg OR specific antihypertensive drug treatment 4. Triglycerides ≥1.70 mmol/L [150 mg/dL] OR lipid lowering treatment 5. HDL-cholesterol ≤1.0 mmol/L [40 mg/dL] (male) and ≤1.3 mmol/L [50 mg/dL] (female) OR lipid lowering treatment
For metALD, the limit of alcohol intake includes an average daily alcohol intake of at least 1. Female 20–50 grams 2. Male 30–60 grams

MASLD is hepatic steatosis involving ≥5% of hepatocytes, detected either by imaging or histology, in individuals with at least one cardiometabolic risk factor e.g., obesity, type 2 diabetes mellitus, hypertension, elevated triglycerides, and with either no alcohol consumption or consumption below stated thresholds.

Metabolic dysfunction–associated steatohepatitis (MASH) is a subset of MASLD, in which there is additional histological evidence of hepatocellular ballooning and lobular inflammation, with or without fibrosis (6). Ultimately, the presence of MASH carries the risk of liver fibrosis, with a consequently higher risk of primary hepatocellular carcinoma.

### 1.1 Shared innate immunity pathways

Innate immunity plays a critical role in MASLD. Liver resident macrophages, known as Kupffer cells, are activated by pathogen-associated molecular patterns (PAMPs), expressed by patients with metabolic syndrome (7) through the binding of toll-like receptor 4 (TLR4). This induces proinflammatory cytokine (e.g., TNF-α, IL-1β and IL-12) and chemokines (e.g., CCL2 and CCL5). Monocyte-derived macrophages (Mo-Ms) are recruited to the liver by activated Kupffer cells. Mo-Ms have a proinflammatory phenotype that drive progression to hepatic steatosis and fibrosis (8). Neutrophils are recruited via activated Kupffer cells, producing IL-6 further promoting liver inflammation. Release of neutrophil granule proteins promote reactive oxygen species promoting hepatocyte inflammation.

The pattern of innate immunity dysregulation via IL-1β production and inflammasome activation is a central convergence across all three conditions. In HS, follicular occlusion release PAMPs and damage-associated molecular patterns (DAMPs) to trigger skin-resident macrophages and dendritic cells to produce IL-1β and TNF-α (1). Similarly, psoriasis demonstrates innate immunity activation through plasmacytoid dendritic cells which produces type 1 interferons and myeloid dendritic cells secreting IL-1β and TNF-α (9). This innate immunity signature is also seen in MASLD that pathogenically drives hepatic inflammation.

Furthermore, neutrophil-mediated inflammation is prominent in these three conditions. HS lesions are neutrophil-rich with formation of neutrophil extracellular trap formation, which can perpetuate inflammation^9^. Psoriasis features a IL-36/neutrophil axis with neutrophil proteases contributing to the inflammatory milieu^10^. As mentioned earlier, recruitment of neutrophils by Kupffer cells is an important driver of hepatic inflammation.

### 1.2 Shared adaptive immunity pathways

HS, psoriasis and MASLD share several key adaptive immunity mechanisms, most notably Th17/Th1 cell activation, IL-17 pathway dysregulation and TNF-α-mediated inflammation.

#### 1.2.1 Th17/Th1 cell activation

Published reports implicate Th1 and Th17 cells in MASLD pathogenesis by skewed balance of elevated proinflammatory Th1 responses (10). The accumulation of Th1 cells with increased hepatic interferon-γ (IFNγ) signalling is seen in MASLD and the genetic ablation of IFNγ in mice protected the mice against MASLD (11). Th17 cells, via amplification of proinflammatory signals driven by Th1 cells, drive further inflammation by inducing the expression of chemokines e.g., CXCL1 and CCL2 that perpetuates the cycle of inflammation through recruitment of neutrophils and Mo-Ms (12).

The Th17/Th1 cell activation pattern is also seen in HS and psoriasis. Activated keratinocytes in the skin of patients with HS release pro-inflammatory cytokines like IL-1β, IL-36α, IL-36β and IL-36γ (13). These cytokines amplify the production of IL-12 and IL-23, which induce adaptive immunity, specifically Th1 and Th17 responses. Psoriasis is a disease with involvement of Th1 and Th17 cells and their secreted cytokines (14). This is supported by the clinical response with biologics targeting specific cytokines of the Th1 (INF-γ and IL-2), Th17 (IL-17A and IL-17F) and Th22 (IL-22) cytokines.

However, critical differences exist in the upstream drivers for HS and psoriasis. In psoriasis, Th17 cells are activated by the IL-23 pathway through myeloid dendritic cells^10^. In contrast, Th17 cells in HS are driven by IL-1β signalling rather than IL-23. This mechanistic distinction explains why IL-23 therapies are more effective in psoriasis.

#### 1.2.2 IL-17 pathway dysregulation

Both HS and MASLD share inflammatory pathways involving dysregulation in T helper 17 (Th17) cells. In HS, IL-23 plays a key role in activating Th17 cells, which in turn produces IL-17 and other pro-inflammatory cytokines. In the liver, increased Th17 cell counts and elevated IL-17 production is observed in patients with MASLD (15). High-fat-fed mice exhibited an increased frequency in IL-17 producing cells in the liver (16). IL-17A inhibiting antibodies prevented MASLD in animal studies (17) and NASH development was prevented in mice lacking IL-17 (18). Interestingly, Th17 cell count decreases significantly 12 months after bariatric surgery (19) in humans. In psoriasis, following activation of plasmacytoid dendritic cells, large amounts of type 1 interferon are produced, leading to the maturation and activation of dermal dendritic cells. The latter cells secrete IL-23, which together with the other cytokines e.g., IL-6, TGF-β, drives naïve T-cells in a Th17 direction.

#### 1.2.3 TNF-α-mediated inflammation

TNF-α serves as a central amplifier in all three conditions, creating a self-perpetuating inflammatory circuit. Increased TNF-α is seen in both skin diseases. TNF-α suppresses adiponectin transcription and downstream production, potentiating insulin resistance. TNF-α is also a major trigger for the production of IL-6, resulting in a proinflammatory state that promotes hepatic steatosis (20). The TNF-α pathway was identified in one study as strongly associated with both psoriasis and MASLD through bioinformatic analysis, with shared upregulation of key genes including MMP9 and CXCL10 (21).

### 1.3 Epigenetic changes

Shared epigenetic pathways between HS and MASLD involves dysregulation of DNA methylation, histone modifications, and non-coding RNAs, molecules that also drive metabolic dysfunction.

In HS, epigenetic changes like DNA methylation and histone modification drive abnormal expression of key inflammatory cytokines and chemokines involved in its shared pathogenesis with MASLD (22). Specifically, microRNAs e.g., miR-155 and miR-132 are pathogenic. miR-155 has an effect on Th17 cell differentiation and function, keratinocyte proliferation and leads to the over-expression of TNF-α in HS and psoriasis patients (23). miR-132 is upregulated during the inflammation phase of wound repair (24). Mice overexpressing miR-132 showed a severe fatty liver phenotype, obesity and hypercholesterolemia. Liver samples from both patients with MASLD and mouse models of hepatic steatosis displayed dramatic increases in miR-132 and varying decreases in miR-132 targets compared with controls (25).

Histone acetylation is another epigenetic mechanism common to the three conditions. They include increased histone acetyltransferase (HAT) activity and altered histone acetylation patterns at inflammatory gene promoters, particularly involving H3K9 acetylation, H3K27 acetylation and H4K16 acetylation that drive pro-inflammatory gene expression, driving chronic inflammation. In one study, patients exhibited a 2.07-fold increase in total HAT activity that correlates with histological steatosis severity (26).

### 1.4 Adipokine imbalance

The skin-liver axis shares a common adipokine dysregulation pattern characterised by decreased adiponectin and increased leptin, resistin and visfatin levels (27).

Adipokines are cell-signalling peptides produced by adipose tissues that regulate metabolism, inflammation and immune response. In obese states, the initial response by the body is to upregulate the production of leptin. This may be initially achieved, but if adiposity continues to increase, leptin fails to compensate for increasing insulin resistance, and paradoxically becomes a proinflammatory phenotype (28). Increased levels of leptin increase hepatic fat accumulation (29). Meta-analysis show increased leptin levels in both HS and psoriasis (30, 31). This explains the common comorbidity of obesity in these populations. In addition, leptin and resistin are increased, and both these adipokines contribute to insulin resistance and metabolic dysfunction (32).

Weight loss can improve HS and psoriasis control, supporting the notion that weight control programmes and medications targeting obesity can be helpful through a reduction in systemic inflammation (33).

### 1.5 Skin-liver axis

The skin-liver axis is driven by several interconnected mechanisms. Systemic low-grade inflammation represents the central link, with both MASLD and inflammatory skin conditions characterised by elevated proinflammatory cytokines including TNF-α, IL-6, IL-17, and IL-23. This cytokine imbalance promotes insulin resistance in multiple tissues (adipose, liver, skeletal muscle), which amplifies hepatic *de novo* lipogenesis and lipid accumulation (34). Exacerbating this is the presence of adipose tissue dysfunction. Dysregulated adipokine fuel inflammation in the liver and skin and the resultant lipotoxicity leads to cellular dysfunction, oxidative stress, cell death in hepatocytes and persistent skin inflammation. The skin-liver axis has important implications for multidisciplinary care. Specific risk phenotypes have been identified for MASLD screening in dermatology patients, including age, metabolic factors, severe inflammatory skin diseases like HS and psoriasis, arthropathy, elevated transaminases (35).

**Figure 1:**
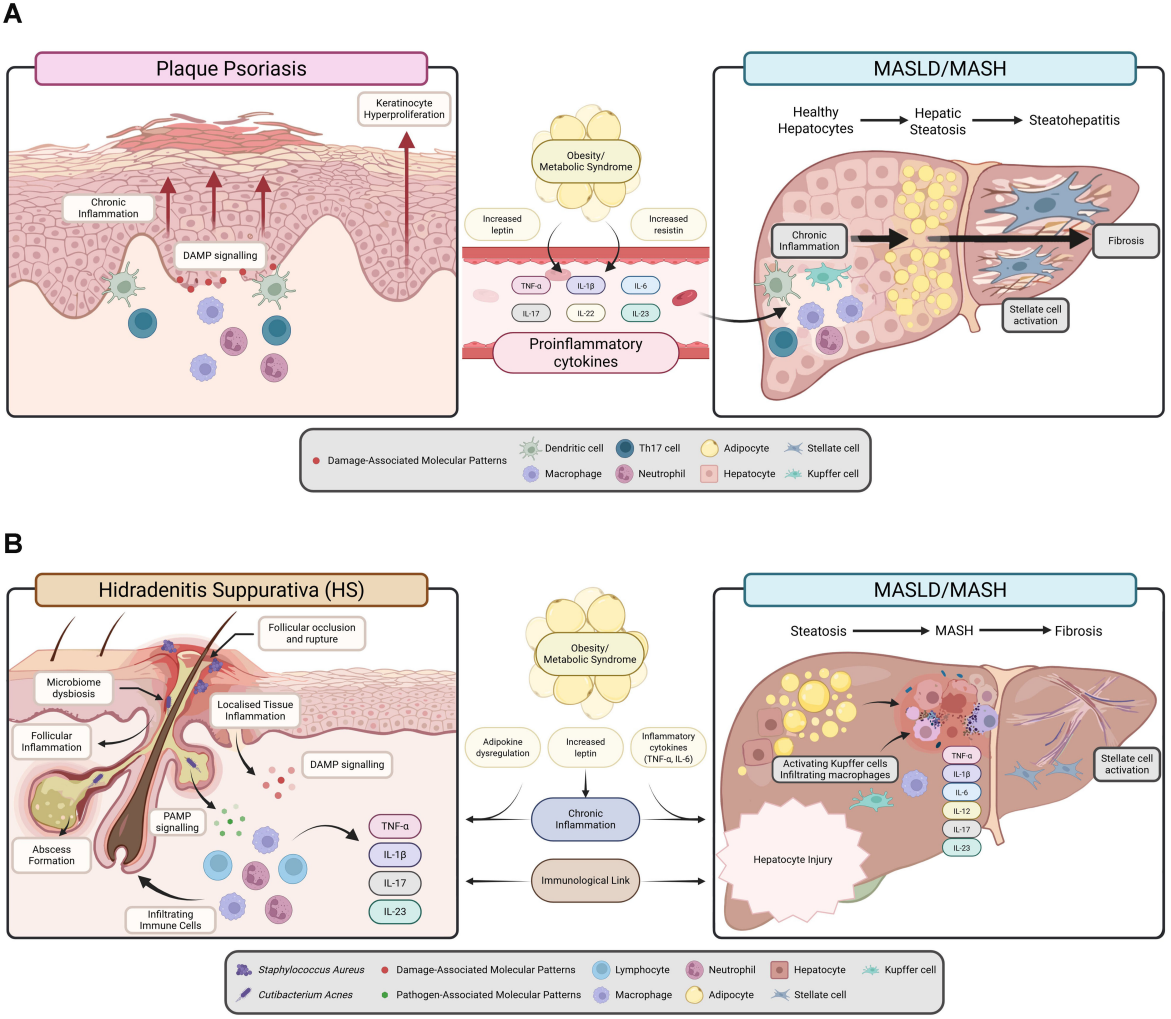
**(A)** Shared immunologic pathways linking plaque psoriasis to hepatic steatosis and fibrosis in MASLD and MASH Tan, W. (2026). Agreement number: OA29M22VHR. https://BioRender.com/raidwwo.  Environmental triggers act on a genetically susceptible individual to amplify cutaneous and systemic inflammation via the IL-23/Th17 axis in plaque psoriasis. TNF-α, IL-1β, IL-6, IL-17 and IL-22 lead to keratinocyte hyperproliferation, immune cell recruitment and cutaneous inflammation. Similar inflammatory and metabolic signals converge in the pathogenesis of MASLD and MASH, where the presence of metabolic syndrome drive insulin resistance, adipokine imbalance, and cytokine-mediated hepatocellular stress. IL-17 promotes hepatic lipid accumulation, including activation of hepatic stellate cells, while cytokines like TNF-α and IL-6 contribute to steatosis, inflammatory amplification and fibrosis. **(B)** Shared immunologic pathways linking hidradenitis suppurativa to hepatic steatosis and fibrosis in MASLD and MASH Tan, W. (2026). Agreement number: OA29M22VHR. https://BioRender.com/raidwwo. In HS, follicular occlusion and rupture in intertriginous skin trigger innate and adaptive immune activation, with immune cells producing cytokines including TNF-α, IL-1β, IL-17, IL-23. This leads to cutaneous inflammation, tissue destruction, and fibrosis. These metabolic and inflammatory signals overlap with MASLD/MASH pathogenesis, where adipose dysfunction, insulin resistance, and pro-inflammatory cytokines drive hepatocellular lipid accumulation, inflammatory cell recruitment, hepatic steatosis, and activation of profibrogenic pathways in hepatic stellate cells, with immune pathways similar to that in plaque psoriasis.

**Figures 1A, B** represent an illustration summarising the integrated immunologic pathways shared among these three diseases.

## 2 Pharmacological measures

### 2.1 Medications for MASLD/MASH of potential benefit in patients with HS and psoriasis

With more therapeutic agents in late-phase clinical trials or are approved for use in patients with MASLD/MASH, dermatologists should be aware of these agents. In a network meta-analysis of 29 randomised controlled trials with more than 9000 patients with biopsy-proven MASH, the top three most effective agents for MASH included pegozafermin, survodutide and tirzepatide. Other useful agents included semaglutide, denifanstat and efruxifermin (36).

Among the therapeutic armamentarium, several agents have demonstrated benefit in the psoriasis and HS population. First, glucagon-like peptide-1 receptor agonists (GLP-1RAs), specifically liraglutide and semaglutide, led to significant reduction in weight and systemic inflammation in HS patients. Notably, improvements in lesion severity and quality of life were reported. The anti-inflammatory effects of GLP-1RAs were attributed to the suppression of key inflammatory pathways involving TNF-α, IL-17, and NF-κB (37). For psoriasis, results are mixed. Case reports and prospective cohort studies suggested benefit of using GLP-1RA for psoriasis, while two small RCTs showed conflicting results, with one study demonstrating skin improvement following GLP-1RAs in patients with T2DM after 12 weeks, and the other showing no effect in glucose-tolerant patients after a shorter timeframe of 8 weeks (38).

Tirzepatide has agonist activity at both the glucose-dependent insulinotropic polypeptide (GIP) and GLP-1 receptors. In the Eli-Lilly phase IIIb trial (TOGETHER-PsA, NCT06588296), a 52-week phase 3b open-label trial evaluating ixekizumab and tirzepatide in 271 adults with active psoriatic arthritis and concomitant obesity with at least one weight-related comorbidity, 31.7% met the primary co-endpoint at week 36 of ACR50 joint response and >10% weight loss compared to ixekizumab monotherapy. The key secondary endpoint of 64% relative increase in the proportion of patients who achieved ACR50 (33.5% of patients vs. 20.4% ixekizumab monotherapy, respectively, p<0.05) was also met.

In the recently published TOGETHER-PsO trial (36), a statistically significant greater proportions of participants who received both Ixekizumab and Tirzepatide met the primary multicomponent end point of complete resolution of psoriasis (PASI 100) and clinically meaningful reduction of body weight (≥10%), as well as the key secondary end point of PASI 100, versus Ixekizumab alone by week 36.

In a retrospective review of safety in combining GLP-1RAs and biologics (adalimumab, dupilumab, risankizumab, secukinumab, ustekinumab) in dermatologic patients, most reported side effects were attributable to an individual, rather than dual therapy. Co-therapy duration was 19 months on average during the study period and discontinuation rate for biologics in dermatology was 18-46%, while that of GLP-1RA was 40-60% during the first two years (39).

Poly-GLP-1 agonists include GLP-1/glucose-dependent insulinotropic polypeptide (GIP) dual agonists (e.g. tirzepatide) which combine GLP-1-mediated glucose-lowering, enhanced insulin secretion and appetite-suppression with GIP’s role in improving insulin resistance and lipid metabolism (40). GLP-1/glucagon (GCG) dual agonists such as survodutide, pair glucagon-induced hepatic fatty acid oxidation and energy expenditure with GLP-1’s ability to offset GCG-induced hyperglycaemia. Retatrutide, triple incretin agonist (GLP-1R/GIPR/CGCR) integrates the actions of all 3 agonists, providing a mechanistically comprehensive approach to metabolic dysfunction and hepatic disease. Oral small molecule formulations of GLP-1 (e.g., semaglutide and orforglipron) are also available. Other drug classes, THR-β agonists (e.g. resmetirom), FGF21-dual agonists modulate lipid metabolism, liver inflammation and fibrosis while FASN inhibitors (e.g. denifanstat) reduce hepatic triglyceride synthesis and may attenuate the progression of liver fibrosis.

These studies suggest that several new therapies for MASLD/MASH, in particular the GLP-1Ras, have a good safety profile when used alongside biologics for psoriasis and HS, particularly in patients with co-existing metabolic comorbidities. Confirmation of their efficacy and safety with psoriasis and HS therapies will require larger trials and registries.

### 2.2 Biologic use in HS and psoriasis with disease-modifying effects in MASLD

Adalimumab is FDA-approved for both moderate-to-severe HS and plaque psoriasis. While there are studies demonstrating improvement in the metabolic profile of these patients (41), there is no direct evidence from randomised controlled trials, post-marketing surveillance, or real-world studies that adalimumab directly improves MASLD or liver-related metabolic dysfunction in HS patients (42). Furthermore, adalimumab carries a liver toxicity rating of B, representing a highly likely cause of clinically apparent liver injury.

A 2025 systematic review showed that IL-17 and IL-23 inhibitors can exert a beneficial effect on reducing the risk of MASLD and liver fibrosis in patients with psoriasis through its shared pathophysiological mechanisms (43). 11 studies assessed the effect of IL-17 inhibitors on MASLD or liver fibrosis; six reported a neutral effect, while five demonstrated improvements in liver tests. Three studies evaluated IL-23 inhibitors; one showed neutral effects, another reported improvement in fibrosis-4 index (FIB-4) scores at 6 months, and the third was still in the recruitment phase.

In the BIOBADERM registry, patients treated with secukinumab had a lower risk of hepatic steatosis as compared to methotrexate (44). There was a reduction in FIB-4 score in patients with elevated indices following 6 months of treatment with secukinumab and ixekizumab (45).

IL-23 inhibitors are approved for treatment of plaque psoriasis, not HS. Regarding the beneficial effects of IL-23 inhibitors, fewer literature exists. Only one study was specifically designed to assess the effect of IL-23 inhibitors on liver outcomes. This retrospective cohort study observed a significant improvement in FIB-4 after 6 months of treatment with IL-23 inhibitors (guselkumab and risankizumab) (46).

Currently, there is an open-label, non-randomized clinical trial recruiting patients to evaluate the effect of guselkumab on MASLD in psoriasis patients, planned to complete in 2028 (47).

### 2.3 Trial exclusion of dermatologic patients

Exclusion criteria in major MASH trials often systematically preclude patients with psoriasis and HS on biologic therapy. The phase 3 survodutide LIVERAGE study (48) by Boehringer Ingelheim prohibits patients with active autoimmune disease on systemic immunosuppressants, effectively barring dermatology patients stabilised on biologics from participating. The ENLIGHTEN-Fibrosis and ENLIGHTEN-Cirrhosis trials require patients on anti-TNF-alpha biologics to be on a stable dose for at least 12 months prior to study commencement (49). The authors hope that modification to existing trials for MASLD and MASH can become dermatology-inclusive so as to benefit these patients.

See **Table 2** for a summary of the drugs discussed.

**Table 2:** Comparative Efficacy and Safety of Therapeutic Classes for Hidradenitis Suppurativa, Psoriasis Vulgaris and MASH^1^.

Class	Drugs	Primary HS Endpoint	Primary PsO Endpoint	Primary MASLD/MASH Endpoint	FDA approval in HS^2^	FDA approval in PsO^2^	Safety Profile	Metabolic Outcomes
TNF-α Inhibitors	Adalimumab, infliximab, etanercept, certolizumab, golimumab	Weekly adalimumab dosing (40 mg subcutaneously) achieved HiSCR-50 at week 12 in 41.8% (PIONEER I) and 58.9% (PIONEER II) of patients, compared to 26.0% and 27.6% for placebo, respectively	Patients treated with infliximab, adalimumab, or etanercept, 76% achieved PASI 75 at week 12: 93% for infliximab, 78% for adalimumab, and 68% for etanercept, using standard induction regimens	No trial data	Only adalimumab is approved	All TNF-α Inhibitors are approved except for golimumab	Weight gain; contraindicated in NYHA III/IV	No consistent impact on total cholesterol, LDL, fasting glucose, blood pressure, or BMI, and no evidence for improvement in metabolic syndrome components
IL-17A Inhibitors	Secukinumab, ixekizumab, brodalumab, bimekizumab	42% of patients receiving secukinumab every 2 weeks achieved HiSCR versus 34% and 31% for placebo, respectively. Efficacy was observed for Bimekizumab in a phase 2 trial which achieved 57.3% HiSCR at week 12 versus 26.1% for placebo. Network meta-analyses confirm that both secukinumab and bimekizumab are associated with significantly higher HiSCR rates than placebo, and are comparable to adalimumab	Ixekizumab reported 81–89% of patients achieve PASI 75 at week 12. Secukinumab achieved 77–82% PASI 75 at week 12. Brodalumab yielded 83–86% PASI 75 at week 12. Bimekizumab, which targets both IL-17A and IL-17F, demonstrates similar or slightly higher PASI 75 rates in real-world cohorts	No trial data	FDA approved (secukinumab, bimekizumab)	IL-17 inhibitors are first-line for moderate-severe psoriasis	Generally well tolerated. Side effects include mucosal candidiasis and injection site reaction	No direct metabolic indications
IL-23 Inhibitors (IL-23/IL-12-23)	Guselkumab risankizumab tildrakizumab, ustekinumab	Interleukin-23 inhibitors have not demonstrated statistically significant improvement in HiSCR rates compared to placebo	For guselkumab and risankizumab, PASI 75 response rates at week 12–16 typically range from 85% to 90% in clinical trials, with guselkumab and risankizumab outperforming tildrakizumab and ustekinumab, as confirmed by network meta-analyses and systematic reviews	No trial data	None	Guselkumab risankizumab tildrakizumab, ustekinumab, icotrokinra	Well-tolerated; infections 5-8%; potential TB reactivation; minimal GI effects	No direct metabolic indication; potential IL-23-mediated modulation of systemic inflammation; Phase 2 NAFLD/PsA trial ongoing (NCT06586281)
IL-1/IL-36 Pathway Inhibitors	Anakinra, spesolimab (IL-36R), lutikizumab (dual IL-1α/β)	Anakinra achieved HiSCR-50 in 78% of patients at week 12, compared to 30% for placebo, but the sample size was small and results at week 24 were not sustained. Lutikizumab (300 mg every 2 weeks) was associated with significantly higher HiSCR-50 and HiSCR-75 response rates compared to placebo, with absolute differences of 24–28% for HiSCR-50 and HiSCR-75, respectively. Spesolimab reduced inflammatory lesions and draining tunnels, with a favourable safety profile, but HiSCR rates were not consistently reported and sample sizes were small	In the pivotal phase 2 randomized trial of spesolimab (an anti-IL-36 receptor monoclonal antibody), 54% of patients treated with spesolimab achieved a GPPGA pustulation subscore of 0 at week 1, compared to 6% with placebo. Additionally, 43% of patients achieved a GPPGA total score of 0 or 1 (clear or almost clear skin) at week 1 versus 11% for placebo, with rapid and sustained improvement in skin symptoms and quality of life observed up to week 12 and beyond	No trial data	None	Spesolimab is FDA approved for generalised pustular psoriasis	IL-1 inhibitors generally well-tolerated; infections 3-5%; potential immunosuppression seen. IL-36 inhibitors are well-tolerated	No direct metabolic effect
JAK/TYK2 Inhibitors	Deucravacitinib, tofacitinib, upadacitinib, baricitinib, povorcitinib	For upadacitinib, 38.3% of patients achieved HiSCR50 at week 12, compared to 25.0% for a prespecified historical placebo rate, and 24% for the in-trial placebo group. For povorcitinib, 48% of patients achieved HiSCR50 and 31% achieved HiSCR75 at week 16, compared to 28% and 17% for placebo, respectively	For tofacitinib, meta-analysis shows PASI 75 rates of 41–59% at week 12 for 10 mg twice daily, with lower rates for 5 mg dosing. For deucravacitinib, PASI 75 response rates at week 12 were 39% (3 mg daily), 69% (3 mg BID), 67% (6 mg BID), and 75% (12 mg daily), compared to 7% for placebo. Upadacitinib achieved PASI 75 in 65% of patients at week 12	No trial data	None	Deucravacitinib is FDA approved for psoriasis	Thromboembolic events; lipid elevation, transaminitis, infections; cardiovascular and malignancy risk in older adults	No primary metabolic label. Dose-dependent lipid elevation requires monitoring
PDE4 Inhibitor	Apremilast	Apremilast achieves HiSCR rates of 53–65% at week 16–24 in moderate-to-severe HS	Apremilast achieved PASI-75 at 40-50% at Week 16 which is weaker than most biologics	No trial data	None	Mild-to-moderate psoriasis as an adjunctive therapy	Diarrhoea, nausea, headache	No primary metabolic label
GLP-1 Receptor Agonists	Semaglutide, liraglutide, dulaglutide	No trial data	No trial data. Case reports demonstrating paradoxical psoriasis flares	GLP-1 RAs are effective in achieving resolution of MASH without worsening of fibrosis	Potential benefit via weight loss and metabolic control but no approval yet	Potential benefit via weight loss and metabolic control but no approval yet	Generally well-tolerated; GLP-1-class effects includes nausea, vomiting, diarrhoea, pancreatitis, sarcopenia	Strong data showing improvement in metabolic profile
GLP-1/GCG Dual Agonist	Survodutide, Tirzepatide	No trial data	In the TOGETHER-PsO trial, among 274 randomised participants, 27.1% of participants simultaneously achieved PASI 100 and a 10% or greater weight reduction with ixekizumab plus tirzepatide vs 5.8% with ixekizumab. Also, 40.6% vs 29.0% of participants achieved PASI 100, 79.9% vs 17.9% simultaneously achieved PASI 75 and a 5% or greater weight reduction, and 69.2% vs 9.1% achieved a 10% or greater weight reduction respectively	Dual incretin agonists have shown greater efficacy. Tirzepatide achieved MASH resolution without worsening fibrosis and ≥1-stage fibrosis improvement in 51–55% of patients, compared to 30% for placebo, and survodutide was superior to placebo for MASH resolution without worsening fibrosis	Potential benefit via weight loss and metabolic control but no approval yet	Potential benefit via weight loss and metabolic control but no approval yet	Dose-dependent heart rate increase (safety signal); peak effect by 6 months. Generally well-tolerated otherwise	Strong data with weight loss and improved metabolic profile
THR-β Agonist	Resmetirom	No trial data	No trial data	MASH Phase 3 n the phase 3 MAESTRO-NASH trial, adults with biopsy-proven MASH and stage F1–F3 fibrosis received resmetirom 80 mg or 100 mg daily for 52 weeks. Both doses resulted in statistically significant improvements in MASH resolution and ≥1-stage fibrosis reduction compared to placebo	Potential benefit via weight loss and metabolic control but no approval yet	Potential benefit via weight loss and metabolic control but no approval yet	LDL cholesterol elevation; pruritus; generally well-tolerated	Modest metabolic effects. Lipid elevation requires monitoring
Pan-PPAR Agonist	Lanifibranor	No trial data	No trial data	Lanifibranor was associated with greater resolution of MASH without worsening of fibrosis and reduction in fibrosis by at least one stage without worsening of MASH compared to placebo in the phase 2b NATIVE trial	Potential benefit via weight loss and metabolic control but no approval yet	Potential benefit via weight loss and metabolic control but no approval yet	Weight gain	Improved insulin resistance, glycated haemoglobin, and plasma lipid levels, but led to moderate weight gain (approximately 2.5%)
FGF21 Analogue	Efruxifermin	No trial data	No trial data	MASH Phase 2b (SYMMETRY, NCT05039450): 24-week interim results showed liver fat reduction ~65% (efruxifermin + GLP-1 RA) vs 10% placebo; liver stiffness reduction 3.0 kPa (combination) vs 1.1 kPa (placebo) withrobust MASH histologic improvement	Potential benefit via weight loss and metabolic control but no approval yet	Potential benefit via weight loss and metabolic control but no approval yet	Increased serum phosphate (~15-20%); hyperphosphatemia monitoring required; otherwise well-tolerated	Enhanced outcomes combined GLP-1 RA (weight loss, improved lipids, enhanced insulin sensitivity)
ACC/DGAT2 Inhibitor Combinations	Clesacostat (ACC) + Ervogastat (DGAT2)	No trial data	No trial data	MASH resolution without worsening fibrosis in 57–63% of patients at 48 weeks, compared to 9% with placebo, with efficacy superior to ervogastat monotherapy and a treatment difference of 0.48–0.54 (90% CI 0.20–0.72) versus placebo. However, improvement in liver fibrosis by at least one stage without worsening of MASH was not significantly greater than placebo in any active treatment group, including the combination	Potential benefit via weight loss and metabolic control but no approval yet	Potential benefit via weight loss and metabolic control but no approval yet	Lipid elevation, inadequate diabetes control common adverse event; mild-moderate	DNL inhibition; modest metabolic benefit; DGAT2 mitigates triglyceride ACC monotherapy
Nonsteroidal farnesoid X receptor (FXR) agonist	Cilofexor, obeticholic acid	No trial data	No trial data	For cilofexor, monotherapy in noncirrhotic NASH demonstrated significant reductions in hepatic steatosis and improvements in liver biochemistry, but did not achieve statistically significant improvement in fibrosis stage or MASH resolution compared to placebo over 24 weeks	None	None	Pruritus, LDL elevation (primary concerns); obeticholic acid program discontinued due to safety/efficacy	Limited primary metabolic indication; bile acid metabolism modulation
SGLT2 Inhibitors	Empagliflozin, dapagliflozin	No trial data	No trial data	In a meta-analysis of 12 phase 2 randomized controlled trials, SGLT2 inhibitors reduced liver fat content (measured by MRI) and lowered serum liver enzyme levels after a median of 24 weeks, indicating improvement in hepatic steatosis and inflammation	None	None	Genital mycotic infections, volume depletion risk and euglycemic DKA	Improved glycaemic control; improved insulin sensitivity; cardiorenal protection; indirect hepatic benefit metabolic improvement)
Combination therapy	Resmetirom and semaglutide	No trial data	No trial data	Combination therapy does not appear to provide additive or synergistic benefit for MASH and MASLD beyond the effect of resmetirom alone, based on current clinical trial data	None	None	Polypharmacy risks; drug-drug interactions and cumulative adverse effects	Weight loss, insulin resistance improvement

## 3 Conclusion

This review article comprehensively covers shared immunological links between MASLD, HS and psoriasis. Given the high prevalence of MASLD in these patients, early disease recognition and intervention is imperative in order to prevent the negative sequelae of progressive liver fibrosis.
